# Health Care Providers’ Acceptance of a Personal Health Record: Cross-sectional Study

**DOI:** 10.2196/31582

**Published:** 2021-10-21

**Authors:** Consuela Cheriece Yousef, Teresa M Salgado, Ali Farooq, Keisha Burnett, Laura E McClelland, Laila Carolina Abu Esba, Hani Solaiman Alhamdan, Sahal Khoshhal, Ibrahim Fahad Aldossary, Omar Anwar Alyas, Jonathan P DeShazo

**Affiliations:** 1 Pharmaceutical Care Department Ministry of National Guard Health Affairs Dammam Saudi Arabia; 2 King Abdullah International Medical Research Center Riyadh Saudi Arabia; 3 King Saud bin Abdul-Aziz University for Health Sciences Riyadh Saudi Arabia; 4 Department of Pharmacotherapy & Outcome Science School of Pharmacy Virginia Commonwealth University Richmond, VA United States; 5 Department of Computing University of Turku Turku Finland; 6 Department of Clinical Laboratory Sciences Cytopathology Practice Program University of Tennessee Health Science Center Memphis, TN United States; 7 Department of Health Administration Virginia Commonwealth University Richmond, VA United States; 8 Pharmaceutical Care Department Ministry of National Guard Health Affairs Riyadh Saudi Arabia; 9 Pharmaceutical Care Services Ministry of National Guard Health Affairs Jeddah Saudi Arabia; 10 Pharmaceutical Care Department Ministry of National Guard Health Affairs Madinah Saudi Arabia; 11 Pharmaceutical Care Department Ministry of National Guard Health Affairs Al Ahsa Saudi Arabia; 12 Royal College of Surgeons in Ireland-Medical University of Bahrain Busaiteen Bahrain

**Keywords:** personal health records, patient portals, Ministry of National Guard Health Affairs, UTAUT, eHealth, Middle East

## Abstract

**Background:**

Personal health records (PHRs) are eHealth tools designed to support patient engagement, patient empowerment, and patient- and person-centered care. Endorsement of a PHR by health care providers (HCPs) facilitates patient acceptance. As health care organizations in the Kingdom of Saudi Arabia begin to adopt PHRs, understanding the perspectives of HCPs is important because it can influence patient adoption. However, no studies evaluated HCPs’ acceptance of PHRs in the Kingdom of Saudi Arabia.

**Objective:**

The aim of this study was to identify predictors of HCPs’ acceptance of PHRs using behavioral intention to recommend as a proxy for adoption.

**Methods:**

This cross-sectional study was conducted among HCPs (physicians, pharmacists, nurses, technicians, others) utilizing a survey based on the Unified Theory of Acceptance and Use of Technology. The main theory constructs of performance expectancy, effort expectancy, social influence, facilitating conditions, and positive attitude were considered independent variables. Behavioral intention was the dependent variable. Age, years of experience, and professional role were tested as moderators between the main theory constructs and behavioral intention using partial least squares structural equation modeling.

**Results:**

Of the 291 participants, 246 were included in the final analysis. Behavioral intention to support PHR use among patients was significantly influenced by performance expectancy (β=.17, *P*=.03) and attitude (β=.61, *P*<.01). No moderating effects were present.

**Conclusions:**

This study identified performance expectancy and attitude as predictors of HCPs’ behavioral intention to recommend PHR to patients. To encourage HCPs to endorse PHRs, health care organizations should involve HCPs in the implementation and provide training on the features available as well as expected benefits. Future studies should be conducted in other contexts and include other potential predictors.

## Introduction

### Overview

A wide range of eHealth technologies has become available over the past 2 decades as countries have introduced eHealth initiatives to support the goals for patient engagement and person-centered care [1]. Legislation around the world advocates for patients to have electronic access to their health information through personal health records (PHRs) [2]. PHRs are an eHealth tool to increase patient engagement and empowerment by allowing individuals to keep track of their personal health information. The Markle Foundation defined PHRs as “an Internet-based set of tools that allows people to access and coordinate their lifelong health information and make appropriate parts of it available to those who need it” [3]. Person-centered care and patient engagement are considered pillars of any high-functioning health care system, and PHRs can contribute to both [4,5]. While various terms have been used interchangeably with PHR in the literature (eg, patient portal, patient web portal, computerized patient portal, patient accessible electronic health record [EHR], tethered PHR, electronic PHR), the broader term of PHR will be used predominantly throughout this paper.

PHR adoption has been associated with a wide range of benefits, including better patient–provider relationships, improvements in patient engagement, better medication adherence, positive health outcomes (eg, blood pressure and glycemic control), and increased organizational efficiencies [6]. As the benefits of PHR adoption are achieved, health care costs potentially decrease as individuals become empowered to take better control of their health and rely less on interactions with the health care system [6]. However, multiple studies have shown low adoption rates [7-9]. Even though the 2009 Health Information Technology for Economic and Clinical Health (HITECH) Act and its Meaningful Use criteria accelerated PHR access in the United States [10], only 15%-30% of patients use PHRs while 90% of health care systems offer them [11]. Outside of the United States, a systematic review showed adoption rates of around 0.13% in the United Kingdom and 5% in other European countries [7].

Various barriers to PHR adoption have been identified [7,9,12,13]. In the systematic review by Niazkhani et al [13], the barriers were characterized as patient demographic factors (eg, age, gender); environment/medical practice (eg, providers’ communication about PHRs, physician resistance); technological (eg, perceived PHR usefulness, perceived PHR complexity); and chronic disease characteristics (eg, patients’ feeling of control over the disease, number of comorbidities). Health care providers’ (HCPs) attitudes are a major contributing factor in patients’ adoption of PHRs [14-16]. HCPs play a key role in supporting and engaging patients through their attitudes, behavior, and endorsement of services [17]. Although studies have shown a high level of patient interest in PHRs [5,18-20], there has been a disconnect between interest and uptake. This is partially due to HCPs’ reticence toward the acceptance and promotion of their use [5,21,22].

Researchers around the world have studied HCPs’ attitudes and perceptions of PHRs. Nazi [22] explored the experiences and perspectives of HCPs (physicians, nurses, and pharmacists) related to patients’ use of the My HealtheVet PHR in the United States and found that many HCPs had limited familiarity with the PHR features, contributing to its underutilization [22]. The author identified the following 8 factors to be key in the implementation, adoption, and use of PHRs: (1) showing the relevance of PHRs; (2) increasing the perceived value by focusing on unique services; (3) providing education and training; (4) integrating PHRs into the existing technology; (5) aligning PHR functions with the workflow; (6) offering incentives to individuals or teams; (7) making information accessible; and (8) supporting asynchronous and bidirectional communication.

A study in Finland, which included a wide range of HCPs (eg, nurses, social workers, dentists, physicians, physical therapists, and psychologists), found that the most important factors influencing HCPs’ support for a national patient portal were expected positive influences on their work, the usability of the portal, and benefits for the patients [17]. However, only few (13%) respondents felt they had received adequate information about the portal. The authors recommended HCPs be informed about PHR benefits to garner their support. In Canada, Wiljer et al [23] endorsed institutional strategies such as “continuous organizational reassurance,” education, and a physician champion to stimulate a paradigm shift to patient-centered care for successful PHR implementation. In a Swedish study of oncology HCPs (nurses and physicians), the authors compared HCPs working in outpatient clinics with those working in primary care units [24]. A greater proportion of HCPs in primary care believed there were benefits of patients using PHRs such as better adherence (50% vs. 35%), greater ability to clarify important information (50% vs. 26%), and improved patient communication (36% vs. 20%) [25].

In the Kingdom of Saudi Arabia, enhancing patient-centered care through patient involvement with technology is an objective of The National Transformation Program, a component of Vision 2030. The Ministry of National Guard Health Affairs (MNGHA) implemented the MNGHA Care PHR in 2018. No studies have evaluated HCPs’ acceptance of PHRs in the country.

The aim of this study was to identify a set of factors that affect the intention to recommend the use of MNGHA Care PHR among HCPs. To promote patient engagement and patient-centered care, a better understanding of how HCPs perceive PHRs is needed.

### Theoretical Background

In 2003, Venkatesh et al [26] developed the Unified Theory of Acceptance and Use of Technology (UTAUT) to provide a comprehensive framework to explain acceptance, intention, and usage of information technology in organizations. It is an integration of 8 theories—theory of reasoned action, technology acceptance model (TAM), motivational model, theory of planned behavior (TPB), combined TAM–TPB, model of personal computer utilization, diffusion of innovation theory, and social cognitive theory [26]. The core constructs of performance expectancy, effort expectancy, social influence, and facilitating conditions directly act on behavioral intention and, ultimately, predict the use of the technology. Gender, age, voluntariness, and experience are moderators in the framework. The model explained approximately 77% of the variance in behavioral intention and 52% of the variance in technology use [26]. Since its development, UTAUT has been used to explain technology acceptance in different user groups in a wide range of contexts with various technologies, strengthening the generalizability [27]. UTAUT has also been used broadly in other health care areas, including telemedicine [28,29], electronic medical/health records [30-34], electronic documentation systems [35], picture archiving and communication systems [36], and health information systems [37,38].

### Research Model and Hypotheses

Most studies have not examined the full UTAUT with the moderation effects but rather the main effects alone, combined with a subset of the moderators, or with new constructs or mechanisms [39]. Venkatesh et al [39] proposed that future research should use UTAUT as the baseline model to transform the theory from static to dynamic. New endogenous mechanisms or new moderation mechanisms are the most common types of extensions [39]. While UTAUT includes the technological dimension (performance expectancy and effort expectancy) and organizational/environmental dimension (social influence and facilitating conditions), the individual dimension is not included. Nonetheless, individual traits (attitude, personal innovativeness, computer self-efficacy) may significantly predict the acceptance of technology [27,40,41]. Constructs representing individual traits are frequently used as endogenous mechanisms to extend UTAUT.

**Figure 1:**
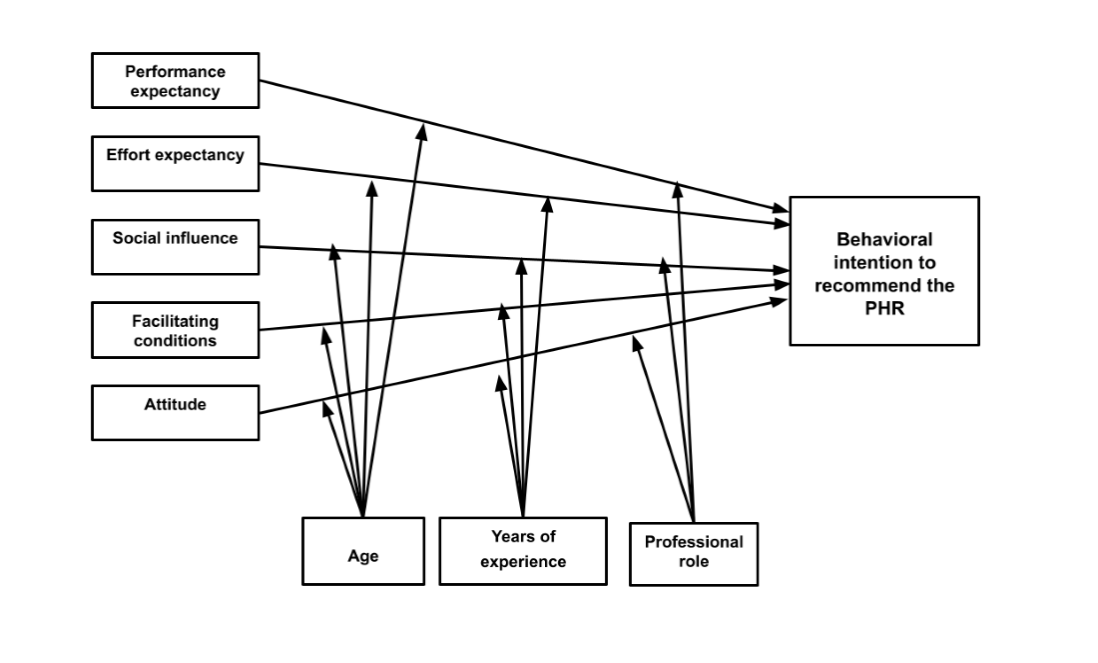
Adapted UTAUT model. PHR: personal health record; UTAUT: Unified Theory of Acceptance and Use of Technology.

The research model for this study includes the 4 core UTAUT constructs: performance expectancy, effort expectancy, social influence, and facilitating conditions (Figure 1). The construct of attitude was added as an individual characteristic. Unlike the original UTAUT model, we did not include behavior in the proposed model because we were unable to objectively assess use. Instead, we measured intention to recommend PHR, using it as a proxy for HCPs’ acceptance. Behavioral intention is frequently a proxy for actual technology adoption in the literature [42-44].

Proposed differences between this model and the original UTAUT model are shown in Table 1. The moderators chosen for this study were age, years of experience, and professional role. Previous literature indicated that age was inversely associated with eHealth adoption. For example, electronic medical record use was inversely associated with physician age [45]. A potential explanation is that, in the initial stages of technology use, older users are believed to be more influenced by experience, and ease of use is more important [26]. Next, years in practice has been associated with acceptance of eHealth [45]. As the number of years since medical school graduation increased, physicians became less likely to accept eHealth technologies [45]. There have also been differences in eHealth acceptance by professional role [45]. Nonphysicians used advanced EHR features less than physicians, and specialists (eg, obstetrician/gynecologists) were less likely to use an EHR in their practices [45]. Voluntariness of use and gender were dropped as moderators in the proposed model. PHR use is not mandatory; therefore voluntariness of use is not relevant [26]. In the health care context, professional role takes precedence over gender and no differences in acceptance by gender were expected [34].

**Table 1:** Original Unified Theory of Acceptance and Use of Technology (UTAUT) versus adapted UTAUT for health care providers.

Construct	Original UTAUT^a^ moderators				Adapted UTAUT moderators		
	Gender	Age	Experience	Voluntariness	Age	Years of experience	Professional role
PE^b^ → BI^c^	✓	✓			✓		✓
EE^d^ → BI	✓	✓	✓		✓	✓	
SI^e^ → BI	✓	✓	✓	✓	✓	✓	✓
BI → Use^f^							
FC^g^ → Use		✓	✓				
FC → BI					✓	✓	
ATT^h^ → BI					✓	✓	✓

^a^UTAUT: Unified Theory of Acceptance and Use of Technology.

^b^PE: performance expectancy.

^c^BI: behavioral intention.

^d^EE: effort expectancy.

^e^SI: social influence.

^f^Use: actual usage.

^g^FC: facilitating conditions.

^h^ATT: attitude.

This study tested the following hypotheses:

- H1: Performance expectancy positively influences behavioral intention to recommend the PHR
- H2: Effort expectancy positively influences behavioral intention to recommend the PHR
- H3: Social influence positively influences behavioral intention to recommend the PHR
- H4: Facilitating conditions positively influence behavioral intention to recommend the PHR
- H5: Attitude positively influences behavioral intention to recommend the PHR
- H6: Age, years of experience, and professional role selectively moderate the relationships between the main constructs and behavioral intention to recommend the PHR

## Methods

### Study Design

A cross-sectional study utilizing a survey was conducted at a large, integrated health care system in the Kingdom of Saudi Arabia. The survey was administered to HCPs across the organization to assess acceptance of the PHR. Since 2018, patients have had access to the MNGHA Care PHR, which includes the following features: scheduling appointments, requesting medical reports and prescription refills, viewing radiology reports, checking laboratory results, and receiving vaccination reminders [46]. Additionally, personal health information such as weight, blood pressure, blood sugar, and exercise details can be uploaded. Finally, MNGHA Care contains links to health education information and a self-assessment feature permitting patients to enter information related to pain control, performance status, and quality of life.

### Setting and Participants

The study population consisted of HCPs from MNGHA hospitals and primary health care centers in Dammam, Riyadh, Jeddah, Madinah, Al Ahsa, and Qassim, including physicians, dentists, pharmacists, nurses, physical and occupational therapists, optometrists, technicians (pharmacy, medical imaging, medical and pathology laboratory, dental), paramedics, and dietitians.

### Instrument and Data Collection

Data were collected using an anonymous self-administered online survey between April and May 2021. The initial version of the survey included 63 items adapted from previously published technology acceptance surveys used in health care in 3 parts [26,47-51]. The first part captured demographic characteristics including age, gender, region, facility type, profession, specialty area (for physicians), years in profession, years at MNGHA, and nationality. The second part contained 4 general PHR questions: (1) Have you heard of MNGHA Care?; (2) Do you have an MNGHA Care account?; (3) Have you used MNGHA Care yourself?; and (4) Have you recommended patients to use MNGHA? This section also included Likert-scale statements associated with PHR acceptance along with an open-ended question and a checklist. The third section related to acceptance of secure messaging and included Likert-scale statements, an open-ended question, and a checklist.

The instrument was built on QuestionPro [52] and pilot tested with 7 HCPs (2 physicians, 3 pharmacists, and 2 nurses) working within MNGHA. The QuestionPro survey link and a cover letter explaining the purpose of the study were emailed to these 7 HCPs to obtain feedback regarding survey length, clarity, and flow of the questionnaire. After comments were compiled, 12 items were removed, and some were modified to improve clarity and to decrease survey length. The final version of the survey included 51 items and was approved by the institutional review boards at the Virginia Commonwealth University and King Abdullah International Medical Research Center.

For this study, the focus was on parts 1 and 2 of the instrument. However, the open-ended question and checklist from part 2 are not included in this paper. Responses to the PHR acceptance items were provided on a 5-point Likert scale from strongly disagree (1) to strongly agree (5). Acceptance was operationalized as the intention to recommend patients use the PHR using the statement “I will probably recommend patients use MNGHA Care in the future” [49].

Performance expectancy was defined as the degree to which the HCP believes a PHR will be beneficial in the health care delivery process [20]. It was measured with the following 4 items:

1. MNGHA Care is a useful tool to help patients feel more involved in their care [47,53].
2. I believe MNGHA Care helps patients to better manage their health [48].
3. MNGHA Care will increase patient satisfaction with their health care [48].
4. MNGHA Care can improve the quality of patient care [51].

Effort expectancy is the degree of ease associated with use of the PHR [20]. It was measured with the following 3 items:

1. Information in MNGHA Care should be easy for our patients to understand [48,53].
2. I believe most patients have the skills needed to use MNGHA Care [47].
3. I think it is not difficult for our patients to learn to use MNGHA Care [47].

Social influence is the degree to which an individual perceives important others believe the PHR should be used [20]. It was measured with the following 2 items:

1. I believe our patients support the use of MNGHA Care [48].
2. In general, the organization has supported the use of MNGHA Care [26].

Facilitating conditions was defined as the degree to which an individual believes an organizational and technical infrastructure exists to support use of the PHR [20]. It was measured with the following 3 items:

1. I have enough information about MNGHA Care [26,48].
2. There is technical help for patients who use MNGHA Care [26].
3. I know the goals of MNGHA Care [26].

Attitude was defined as positive feelings related to patients using the PHR [54]. It was measured with the following 4 items:

1. MNGHA Care is a valuable tool [26,47].
2. It is a good idea for patients to use MNGHA Care [26,47].
3. MNGHA Care is a positive advancement in this digital age [47].
4. I believe MNGHA Care will be used by many patients [47].

Although behavioral intention and social influence used less than 3 items, partial least squares structural equation modeling (PLS) supports using single-item measures [55] and earlier research using PLS has used less than 3 items for measuring constructs [56,57].

### Sampling

A snowball and convenience sampling strategy was used to recruit HCPs from across the organization. HCPs were initially recruited through the hospital’s email list in combination with WhatsApp as it is a widely used social media platform for professional communication. They were asked to forward the survey to other HCPs. Follow-up reminders were also sent out. The target sample size for this study was 200 HCPs, which has been considered a fair sample size for statistical analysis with structural equation modeling [58]. To encourage participation, there was a random drawing for twenty five 37.5 Saudi Arabian Riyal (US $10) Amazon gift cards.

### Statistical Analyses

Descriptive statistics were analyzed using SPSS version 25 (IBM) [59]. PLS was used to test the research model using SmartPLS version 3.0 [60]. The advantage of PLS is the ability to estimate complex research models without distributional assumptions [61]. Compared with traditional SEM, PLS has greater statistical power, which means that there is a higher likelihood of identifying significant relationships if they are actually present in the population [61]. Furthermore, PLS has been widely used in empirical studies of technology acceptance, including with UTAUT [26,27,34] and with PHR acceptance [50,62]. To test the research model, a measurement model was used to evaluate construct reliability, indicator reliability, convergent validity, and discriminant validity. A structural model was tested after ensuring reliability and validity.

## Results

### Demographic Characteristics

Overall, 291 HCPs participated in the survey. However, after removing the data for missing values, a usable sample of 246 was used for further analysis. Table 2 presents the demographic characteristics. Most were 40-49 years old (95/246, 38.6%), female (158/246, 64.2%), non-Saudi (132/246, 53.7%), nurses (106/246, 43.1%), in Riyadh (81/246, 32.9%), over 10 years of experience (167/246, 67.9%) and over 10 years at MNGHA (128/246, 52.0%), and hospital based (228/246, 92.7%).

**Table 2:** Demographic characteristics (N=246).

Variables		Values, n (%)
**Age**		
	20-29 years	37 (15.0)
	30-39 years	77 (31.3)
	40-49 years	95 (38.6)
	50 years and above	37 (15.0)
**Gender**		
	Male	88 (35.8)
	Female	158 (64.2)
**Nationality**		
	Saudi	114 (46.3)
	Non-Saudi	132 (53.7)
**Health care provider**		
	Physician	40 (16.3)
	Pharmacist	57 (23.2)
	Nurse	106 (43.1)
	Technician	33 (13.4)
	Other	10 (4.1)
**Years in profession**		
	Less than 5 years	33 (13.4)
	5-10 years	46 (18.7)
	Greater than 10 years	167 (67.9)
**Years working at MNGHA^a^**		
	<1 year	13 (5.3)
	1-4 years	40 (16.3)
	5-10 years	65 (26.4)
	>10 years	128 (52.0)
**Location**		
	Dammam	46 (18.7)
	Madinah	35 (14.2)
	Al Ahsa	51 (20.7)
	Jeddah	33 (13.4)
	Riyadh	81 (32.9)
**Type of facility**		
	Hospital	228 (92.7)
	Primary health care clinic	18 (7.3)

^a^MNGHA: Ministry of National Guard Health Affairs.

### General PHR Use Characteristics

The majority of HCPs were aware of MNGHA Care (225/246, 91.5%), had an account (213/246, 86.6%), used MNGHA Care (202/246, 82.1%), and recommended it to patients (198/246, 80.5%).

### Measurement Model

The measurement model testing results are summarized in Table 3. After removing missing data, the usable sample for hypothesis testing was 246. The variance inflation factor of all items was below the threshold of 5, showing no evidence of multicollinearity. Item loadings were all above 0.40 and in the range of 0.70-0.93. Composite reliability was above the threshold of 0.70, showing good internal consistency. Moreover, the average variance extracted (AVE) of the constructs were greater than 0.50 and in the range of 0.55-0.81, indicating convergent validity.

Discriminant validity was tested using the Fornell–Larcker criterion. The square roots of the corresponding AVE are shown in italics, with each construct’s AVE higher than its highest correlation with any other construct (Table 4). Results in Tables 3 and 4 provide evidence of the validity and reliability of the constructs used in the model.

**Table 3:** Measurement model statistics.

Construct and items		Mean	SD	VIF^a^	Loadings	CR^b^	AVE^c^
**Performance expectancy (PE)**		4.09	0.73			0.95	0.81
	PE1			2.526	0.87		
	PE2			3.792	0.92		
	PE3			3.711	0.92		
	PE4			3.462	0.90		
**Effort expectancy (EE)**		3.75	0.67			0.79	0.55
	EE1			1.099	0.81		
	EE2			1.473	0.70		
	EE3			1.465	0.72		
**Facilitating conditions (FC)**		3.60	0.78			0.88	0.71
	FC1			1.756	0.85		
	FC2			1.547	0.77		
	FC3			2.023	0.90		
**Social influence (SI)**		3.82	0.69			0.85	0.74
	SI1			1.3	0.84		
	SI2			1.3	0.88		
**Attitude (ATT)**		4.08	0.63			0.94	0.80
	ATT1			4.171	0.93		
	ATT2			3.603	0.92		
	ATT3			3.486	0.91		
	ATT4			2.029	0.83		
**Behavioral intention (BI)**							
	BI	4.18	0.68		1	1	1

^a^VIF: variance inflation factor.

^b^CR: composite reliability.

^c^AVE: average variance extracted.

**Table 4:** Discriminant validity of the constructs.^a^

Constructs	1	2	3	4	5
Attitude	*0.896*				
Effort expectancy	0.697	*0.742*			
Facilitating conditions	0.596	0.570	*0.843*		
Performance expectancy	0.742	0.708	0.527	*0.901*	
Social influence	0.646	0.671	0.645	0.602	*0.860*

^a^Square roots of the corresponding average variance extracted are shown in italics.

### Structural Model

**Figure 2:**
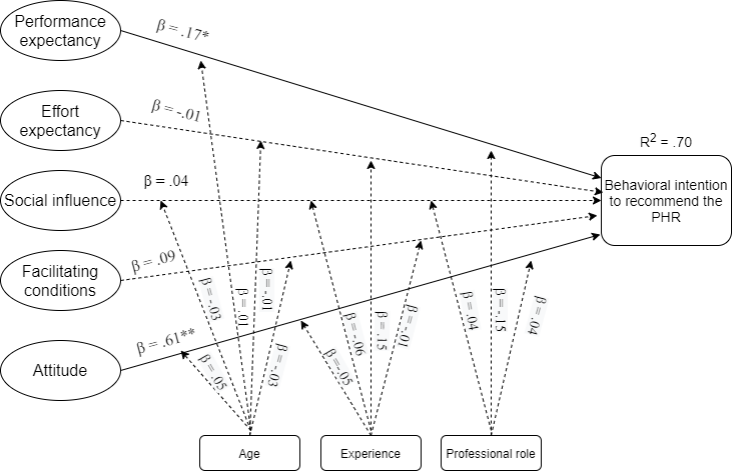
Structural model showing path coefficients (β) and coefficient of determination (*R*^2^) (*P<.05, **P<.01). PHR: personal health record.

The *R*^2^ was used to assess the structural model. Overall, the model explained 70% of the variance in the intention to recommend the PHR among HCPs (Figure 2). Table 5 presents the structural model results, while Table 6 presents the test for moderating effects.

**Table 5:** Structural model results.

Structural model	β	*t*-statistics^a^ (*df*)	*P* value	*f* ^2^
PE^b^ → BI^c^	.17	2.132 (499)	.03^d^	0.035
EE^e^ → BI	–.01	0.166 (499)	.87	0
SI^f^ → BI	.04	0.473 (499)	.63	0.002
FC^g^ → BI	.09	1.241 (499)	.21	0.013
ATT^h^ → BI	.61	6.385 (499)	<.01	0.369

^a^Two tailed.

^b^PE: performance expectancy.

^c^BI: intention to recommend PHR.

^d^P<.05.

^e^EE: effort expectancy.

^f^SI: social influence.

^g^FC: facilitating conditions.

^h^ATT: attitude.

**Table 6:** Moderation analysis results.

Analysis		β	*t*-statistics^a^ (*df*)	*P* value	*f* ^2^
**Moderation of age**					
	PE^b^ × AGE^c^ → BI^d^	.01	0.118 (499)	.91	0
	EE^e^ × AGE → BI	–.01	0.159 (499)	.87	0
	FC^f^ × AGE → BI	–.03	0.360 (499)	.72	0.001
	SI^g^ × AGE → BI	.05	0.633 (499)	.53	0.003
	ATT^h^ × AGE → BI	–.03	0.307 (499)	.76	0.001
**Moderation of experience**					
	EE × EXP^i^ → BI	.15	1.688 (499)	.09	0.016
	SI × EXP → BI	–.06	0.609 (499)	.54	0.003
	FC × EXP → BI	–.01	0.205 (499)	.84	0
	ATT × EXP → BI	–.05	0.597 (499)	.55	0.003
**Moderation of professional role**					
	PE × HCP^j^ → BI	–.15	1.598 (499)	.11	0.023
	SI × HCP → BI	.04	0.620 (499)	.54	0.003
	ATT × HCP → BI	.04	0.441 (499)	.66	0.002

^a^Two tailed.

^b^PE: performance expectancy.

^c^AGE: age.

^d^BI: intention to recommend PHR.

^e^EE: effort expectancy.

^f^FC: facilitating conditions.

^g^SI: social influence.

^h^ATT: attitude.

^i^EXP: experience.

^j^HCP: health care provider.

## Discussion

### Principal Findings

To the best of our knowledge, this is the first study to examine factors that influence HCPs’ intention to recommend PHRs to patients in the Kingdom of Saudi Arabia. Prior studies in the country evaluated the challenges in implementing PHRs and identified HCP resistance as a barrier [63,64]. Although HCPs are not the primary users of PHRs, their endorsement can positively influence patient engagement with this technology [12]. While some providers find promoting the PHR to be an additional burden, those providers who present a PHR to their patients as a tool to supplement their care can facilitate patient adoption [9]. Our study found a high level of awareness among HCPs, with 88.2% (217/246) having an account and 82.1% (202/246) recommending patients use the PHR. In our previous study in patients, HCPs and hospital staff were primarily responsible for recommending the PHR in 58.7% of patients who reported using MNGHA Care [18].

Predictors of patient adoption of PHRs may differ from those that affect HCPs to endorse a PHR [7,12]. Therefore, the research model for HCPs was slightly different from the one used for patients [18]. The proposed theoretical model explained 70% of the variance in HCPs’ behavioral intention to recommend PHRs to patients. Performance expectancy and attitude were significantly associated with behavioral intention to recommend the PHR. Much of the literature has shown performance expectancy as the strongest predictor of intention to use technology among HCPs [30,38,47]. In patient and consumer studies of PHRs, performance expectancy has also been a positive predictor of adoption [50,65-67]. However, the attitude was the strongest predictor of behavioral intention in our study. Other studies on PHR adoption have also found attitude to be an important predictor [47,51].

Our findings did not support the hypothesis that age, years of experience, and professional role moderate behavioral intention. Several studies have shown that older and more experienced HCPs are more resistant to health information technology and are less comfortable with using technology [35]. Physicians also have been found to be less enthusiastic about the introduction of eHealth services [33]. In our study, most had over 10 years of experience as an HCP (190/289, 65.7%) and more than 10 years in MNGHA (149/289, 51.6%). Furthermore, while Moll and Cajander [25] found differences in attitudes of HCPs who worked in primary care units compared with outpatient clinics, most HCPs in this study were from the hospital (265/284, 93.3%), limiting the ability to draw comparisons.

### Implications for Theory

This research adds to the literature on HCPs’ acceptance of PHR using an adapted UTAUT model. To our knowledge, this is the first study to extend UTAUT with the construct of attitude in the context of HCPs’ acceptance of PHR. Only few studies evaluating HCPs’ acceptance of PHRs have used theory [68]. This study revealed that the adapted UTAUT model was a good predictive model of HCPs’ behavioral intention to recommend PHR use. While our model found that performance expectancy and attitude individually influence behavioral intention, it may also be the case that attitude mediates the relationship between performance expectancy and behavioral intention, as proposed by Dwivedi et al [40].

The original UTAUT explained 76% of the variance in behavioral intention. No studies on PHR adoption have used the original UTAUT model [50,65,66,69,70]. The advantage of the adapted model is a similar predictive power while parsimoniously eliminating the construct “use behavior” and the moderator “voluntariness” in the original model. Although the model explained 70% of the variance in behavioral intention and provided support for the proposed theoretical model, other factors may be important for HCPs’ acceptance of PHR. In the health care setting, UTAUT has been criticized for its focus on general technology acceptance factors and the inability to completely explain health information technology adoption [71]. Therefore, it is recommended that UTAUT be adapted to fit the health care context by incorporating health behavior theories, privacy and security issues, and negative factors that inhibit technology adoption [71].

### Implications for Practice

This study provides practical contributions based on the proposed relationships and supports the need to focus on strategies to enhance perceived usefulness and a positive attitude toward the PHR in HCPs. While some patients view self-management as a burden and prefer the status quo [9], others will respond to HCP’s endorsement of the use of PHRs. Several studies identified HCP recommendation as an important factor in patients’ choosing to use PHRs [2,22,23,72,73]. Lyles et al [11] found one-on-one training to be the most effective intervention in PHR implementation in the United States. Providing short educational sessions to individuals or teams can facilitate acceptance among HCPs [48]. These training sessions could be conducted by each department. Training HCPs on the features available supports successful implementation by increasing skills and knowledge. Campaigns can also be directed at promoting awareness among HCPs. Through these interventions, HCPs will perceive the usefulness of PHRs and develop more positive attitudes regarding the benefits. Consequently, they will be more inclined to recommend PHRs to patients. Through their interactions with HCPs, patients will perceive PHRs as useful and are more likely to adopt them [66].

### Limitations

There are several limitations to this study. While cross-sectional studies are useful for examining associations, a causal relationship cannot be established [74]. Snowball and convenience sampling, both nonprobability sampling strategies, were used to select participants, limiting generalizability; however, participants from multiple sites were selected to attain good representation across MNGHA. Self-administered online surveys are associated with various biases, including social desirability response bias, self-selection, and nonresponse bias [75]. To minimize social desirability response bias, participants had the option of not answering any question that made them uncomfortable. To minimize nonresponse bias, HCPs were contacted multiple times and offered an incentive to encourage a high response rate.

### Recommendations for Future Research

Future studies should evaluate the proposed model in other contexts. This study involved a large integrated health care organization. Research in other organizations within the country and in this part of the world will increase the generalizability of our findings. Research should also be conducted in individual HCP groups. Differences in PHR acceptance have been observed based on a variety of characteristics, including age, gender, professional role, and practice setting. Future researchers should focus on HCP group–specific interventions. Finally, while this study used an open-ended question and checklist (analyzed separately) to achieve greater depth, one-on-one interviews would provide valuable data on the motivation of HCPs and nuances within this context.

### Conclusion

This study examined factors affecting HCPs’ behavioral intention to recommend PHRs to patients in the Kingdom of Saudi Arabia. The proposed model accounted for 70% of the variance in behavioral intention, indicating significant predictive power. Performance expectancy and attitude were significant predictors of HCPs’ behavioral intention to support PHR use. Our results suggest that health care organizations should focus on strategies associated with these factors to improve HCP support and decrease barriers to patient use of PHRs. Future research should test this model and explore other predictors in order to develop successful interventions to encourage the adoption and continued use of the PHR among patients.

## Abbreviations

EHR: electronic health record
HCP: health care provider
HITECH: Health Information Technology for Economic and Clinical Health
MNGHA: Ministry of National Guard Health Affairs
PHR: personal health record
TAM: technology acceptance model
TPB: theory of planned behavior
UTAUT: Unified Theory of Acceptance and Use of Technology
